# Tube-in-tube for pancreatic fistula after pediatric pancreatic surgery: a case report

**DOI:** 10.1093/gastro/goae063

**Published:** 2024-07-02

**Authors:** Yufeng Li, Yinghui Song, Chenlin Jiang, Yuhang Li, Chenji Tang, Sulai Liu

**Affiliations:** Department of Hepatobiliary Surgery, The First Affiliated Hospital of Hunan Normal University, Hunan Provincial People’s Hospital, Changsha, Hunan, P. R. China; Central Laboratory of Hunan Provincial People’s Hospital (The First Affiliated Hospital of Hunan Normal University), Changsha, Hunan, P. R. China; Hunan Provincial Key Laboratory of Biliary Disease Prevention and Treatment, Changsha, Hunan, P. R. China; Department of Hepatobiliary Surgery, The First Affiliated Hospital of Hunan Normal University, Hunan Provincial People’s Hospital, Changsha, Hunan, P. R. China; Department of Hepatobiliary Surgery, The First Affiliated Hospital of Hunan Normal University, Hunan Provincial People’s Hospital, Changsha, Hunan, P. R. China; Department of Hepatobiliary Surgery, The First Affiliated Hospital of Hunan Normal University, Hunan Provincial People’s Hospital, Changsha, Hunan, P. R. China; Central Laboratory of Hunan Provincial People’s Hospital (The First Affiliated Hospital of Hunan Normal University), Changsha, Hunan, P. R. China; Hunan Provincial Key Laboratory of Biliary Disease Prevention and Treatment, Changsha, Hunan, P. R. China

## Introduction

The pancreas is a retroperitoneal organ with an insidious location, making pancreatic injuries relatively rare [1]. Pancreatic injury is the fourth most common type of organ injury in children with abdominal trauma, following injuries to the spleen, kidney, and liver [2]. In several common classifications, pancreatic transection injuries are considered one of the most severe types of pancreatic injury [3, 4]. The surgical treatment for pancreatic transection in children is usually distal pancreatectomy, pancreaticojejunostomy, or pancreaticoduodenectomy, depending on the site and degree of injury. Postoperative pancreatic fistula (POPF) is a frequent complication following pancreatic transection injury, which significantly increases the mortality rate of patients. In this study, we inserted an external pancreatic duct stent into a T-tube to reduce the incidence of POPF due to transection of the pediatric pancreas and this method achieved good results.

## Case presentation

The patient was a 5-year-old girl who presented severe abdominal pain 12 hours after blunt abdominal trauma and was admitted to Hunan Provincial People’s Hospital. The patient had no relevant medical history. The patient’s consciousness was clear, with a temperature of 37.6°C, a pulse of 120 beats/min, a respiration rate of 35 breaths/min, a blood pressure of 124/75 mmHg, and an SPO_2_ of 96%. Abdominal computed tomography (CT) revealed transverse injury to the neck of the pancreas with hematoma formation. At admission, her hematological results showed a significant increase in serum amylase and lipase, with amylase levels at 443.0 U/L and lipase levels at 440 U/L.

We made a 12-cm incision in the middle of the patient’s upper abdomen and found that the pancreas neck was transected, leading to injury and hematoma formation, with the caudal portion of the pancreas exhibiting edema. The hematoma and necrotic tissue were removed and the severed end of the head of the pancreas was closed using Prolene sutures. The necrotic tissue of the body and tail of the pancreas was resected and an epidural catheter was placed in the pancreatic duct to act as an external stent, which was then placed in a 16-gauge T-tube (5.28 mm in diameter). Roux-en-Y anastomosis of the jejunum was performed on the pancreatic stump.

The long arm of the T-tube (with a built-in epidural catheter) was extracted on the side of the stump of the jejunal loop. The transverse arm of the T-tube was fixed to the pancreatic parenchyma using absorbable sutures. The decompressed T-tube was discharged through the left anterior abdominal wall and a drainage tube was placed behind the pancreaticojejunal anastomosis through the right anterior abdominal wall.

**Figure 1.**
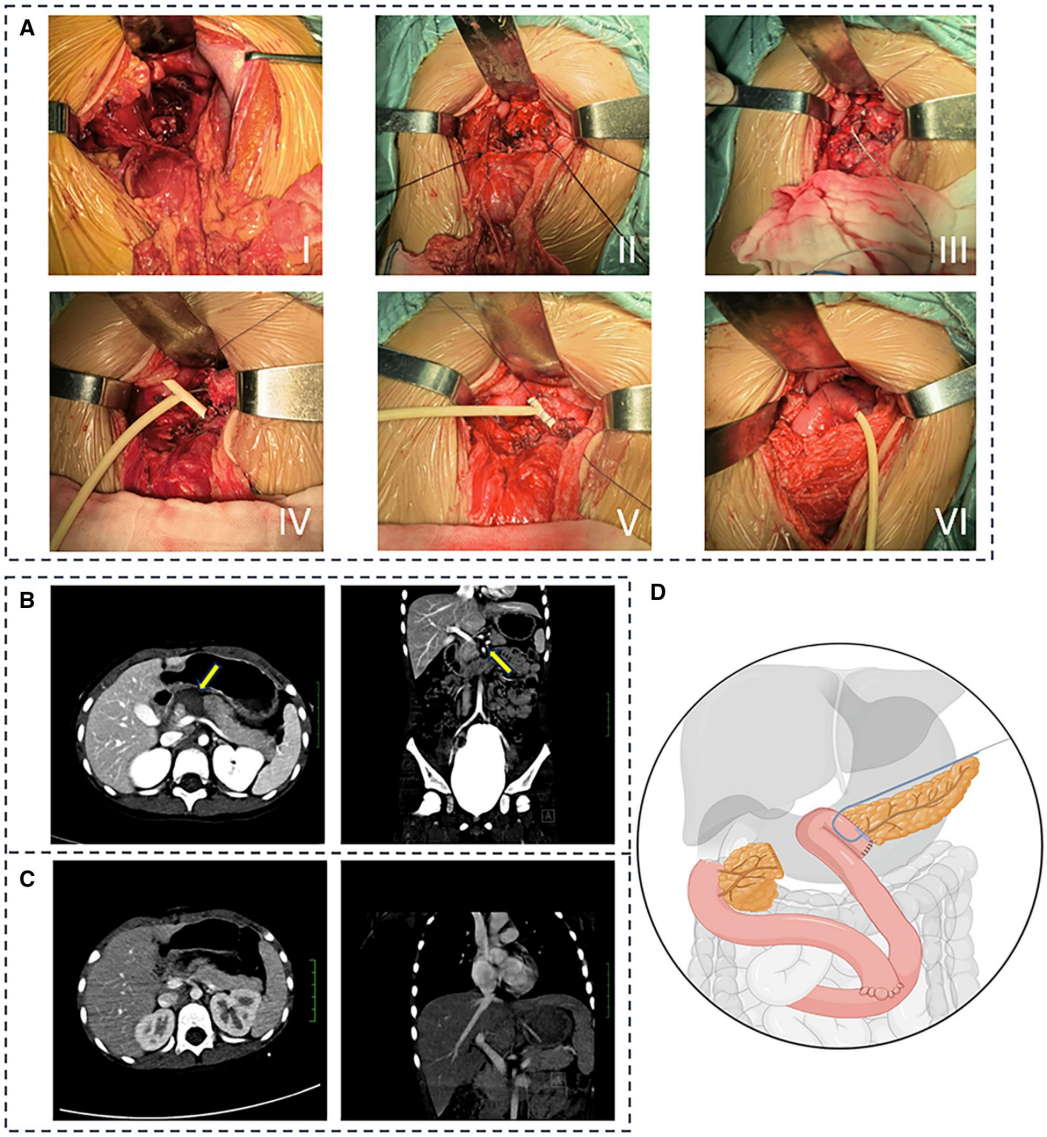
The clinical data of the patients and schematic diagram of the surgery. (A) I. The neck of the pancreas was transected and bleeding occurred. II: Transected pancreatic neck. III: An epidural catheter was placed into the pancreatic duct. IV: The epidural catheter was placed into the T-tube. V: The T-tube was sutured to the pancreas using absorbable wire. VI: The pancreas and jejunal ansae were anastomosed using Chen’s U-stitch. (B) Preoperative abdominal CT shows pancreatic neck transection and local hematoma formation (arrowhead). (C) Postoperative abdominal CT. (D) Schematic diagram of the surgery.

Intraoperative vital signs were stable and the surgery proceeded without complications for the patient. After surgery, the patient fasted for 5 days and received total parenteral nutrition. Repeat CT showed no obvious postoperative complications (Figure 1). The patient was discharged with an external stent and T-tube after 14 days postoperatively. The external stent and T-tube were placed for 60 days after the operation and then the external stent and T-tube were directly removed. The patient was followed up for 7 months and there was no stenosis/obstruction, pancreatic fistula, or bleeding after removal of the stent.

## Discussion

Pediatric pancreatic injuries are rare, occurring in <1% of cases [5, 6]. Blunt trauma is the most common cause. Patients with low-grade pancreatic injuries (Grades I–II) can be treated non-surgically, whereas treatment of high-grade (Grades III–V) patients with nonsurgical or surgical treatment is controversial [7]. Kopljar *et al.* reported a meta-analysis of initial nonsurgical vs initial surgical treatment in children with blunt pancreatic injuries [8]. The study found that the patients with Grade III–V pancreatic injuries revealed a significantly greater risk of pseudocyst formation in the non-operative management group (odds ratio 12.46, 95% confidence interval 4.60 to 33.77, *P *<* *0.00001) and an unchanged risk of pancreatic fistula formation. Therefore, the authors concluded that these patients would not benefit from nonsurgical treatment. For this child, the vital signs and imaging were evaluated on admission and, after multidisciplinary treatment consultation, the child was considered to have a Grade IV pancreatic injury, the efficacy of conservative treatment was not clear, and surgical treatment was selected.

According to the Western Trauma Association, patients with high-grade pancreatic injury can undergo pancreatectomy or pancreaticoduodenectomy, but the above surgical methods have a greater risk of POPF [9]. The proximal pancreatic stump suturing and distal pancreatic jejunostomy were adopted at our center. To minimize the incidence of POPF, keep the pancreatic duct open, and prevent stenosis, a stent is usually chosen at the pancreaticoenteric anastomosis site [10]. Pancreatic duct stenting is associated with a variety of serious complications, such as pancreatitis, duodenal perforation, hemorrhage, stent fracture, and obstruction, which are often caused by different stent choices, stent destruction, and inappropriate placement. There are two commonly used options for stenting: external pancreatic duct stents and internal pancreatic duct stents. In terms of stent selection, a meta-analysis has shown that external stenting reduces the incidence of POPF compared with internal stenting [11]. In terms of stent damage and improper placement, in our experience, it is difficult to place an individualized external pancreatic duct stent due to the limitations of the shape of the stent and the influence after pancreaticojejunostomy. More importantly, the stent cannot be fixed well. Therefore, the above factors may lead to the occurrence of complications.

We innovatively placed an external pancreatic duct stent into the T-tube and secured the transverse arm of the T-tube to the pancreatic parenchyma using absorbable sutures. The long arm of the T-tube was then withdrawn from the stump side of the jejunal collaterals to make it more effective at supporting the pancreaticoenteric anastomosis. More importantly, the placement of the extrapancreatic stent into the T-tube not only effectively prevents the extrapancreatic stent from breaking, but also strengthens the position of the extrapancreatic stent. At the same time, it avoids pancreatic fluid erosion and reduces pancreatic duct obstruction caused by local pressure on the pancreaticojejunal anastomosis, which largely reduces the complications associated with pancreatic duct stenting. Due to the soft nature of the T-tube and the low resistance of the shortened cross arm, the drain and T-tube can be removed directly without causing trauma to the pancreaticojejunal anastomosis. This innovative procedure provides a new surgical approach for patients with high-grade pancreatic injuries, which not only allows greater preservation of pancreatic tissue, but also prevents POPF and effectively reduces complications associated with pancreatic duct stent placement.

In conclusion, the use of absorbable sutures to fix the T-tube with a built-in pancreatic duct stent to the pancreaticojejunal anastomosis can effectively prevent POPF in patients with pancreatic transection injury.

## Authors’ Contributions

Y.L. and C.J. helped to collect clinical data; Y.S. and Y.L. contributed significantly to manuscript preparation; C.T. and S.L. helped with surgical planning.
